# Supplementary material for: Cellular modifiers of TDP-43 phase transition and cytoplasmic aggregation
Source: eLife. 2026 Sep 8;15:RP110172. doi: 10.7554/eLife.110172 (PMC13553071; doi:10.7554/eLife.110172)

Figure 1-figure supplement 1

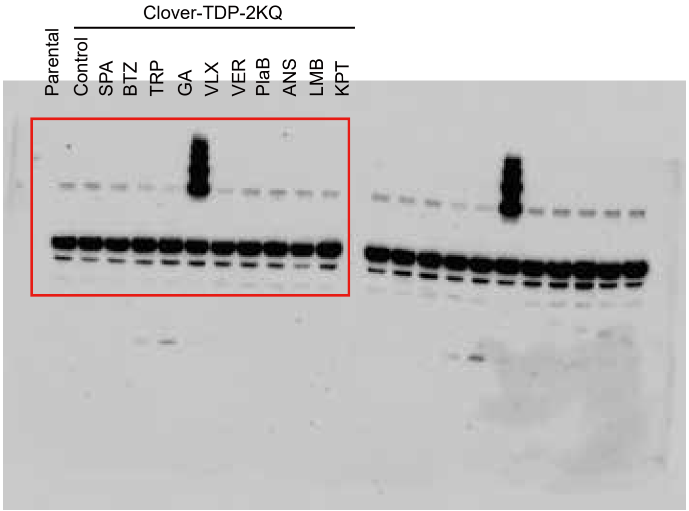

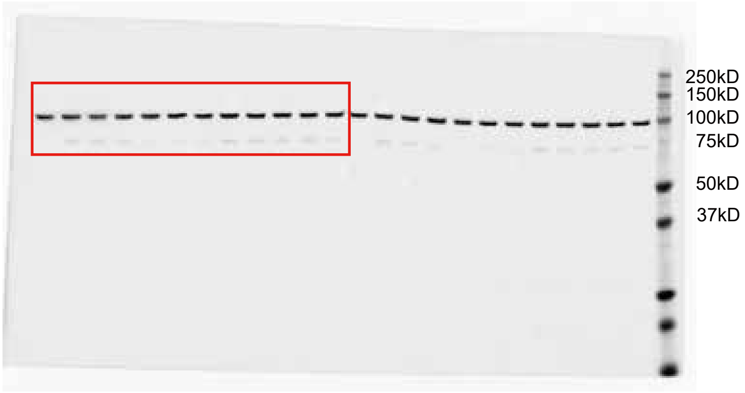

Supplement: Supplementary file 3 [file elife-110172-fig1-figsupp1-data2.zip › Supplement Figure 1 labeled gels/Figure 1-supplement figure 1.pdf]
