# Supplementary material for: Cellular modifiers of TDP-43 phase transition and cytoplasmic aggregation
Source: eLife. 2026 Sep 8;15:RP110172. doi: 10.7554/eLife.110172 (PMC13553071; doi:10.7554/eLife.110172)
Supplement: Peer review history

# Editor report: eLife Assessment

**DOI:** 10.7554/eLife.110172.4.sa0

**Authors:** Paul Donlin-Asp

**Affiliations:** University of Edinburgh United Kingdom

**Keywords:** Solid, Valuable

This revised study offers **valuable** insights into how nuclear export influences protein condensates and TDP-43 phase behaviour. The findings are **solid** and highlight several noteworthy observations for the field, such as RNA-dependent stabilisation of TDP-43 condensates and the inhibition of nuclear export in an ALS organoid model. The work suggests a possible mechanistic link between nuclear export and TDP-43 aggregation in ALS/FTD; however, the exact mechanistic connection remains unclear, as much of the evidence is based on indirect observations within sensitised model systems. Although the authors carefully acknowledge these limitations and moderate their conclusions, further validation in more physiologically relevant models will be necessary to demonstrate a direct causal role of nuclear export in regulating pathological TDP-43 aggregation.

# Referee report: Reviewer #1 (Public review)

**DOI:** 10.7554/eLife.110172.4.sa1

[Editors' note: this version has been assessed by the Reviewing Editor without further input from the original reviewers. The authors have addressed the comments raised in the previous round of review.]

This revised manuscript represents a partial response to the concerns raised in the first round of review. The authors have made one genuine mechanistic addition in the form of the semi-permeabilized cell reconstitution assay, removed the most overreaching conclusions regarding the contribution of cytoplasmic TDP-43 aggregation to disease, and made several minor presentational improvements. However, the central weaknesses of the original submission remain substantially unaddressed. The exclusive reliance on non-physiological TDP-43 variants, the incompletely resolved mechanism linking XPO1 to TDP-43 phase behavior, and the limited organoid validation continue to limit confidence in the major claims. The authors have, in several instances, responded by removing contested data rather than by providing the additional evidence that was requested.

(1) The justification for the 2KQ acetylation-mimetic system remains inadequate.

The authors respond to the concern about the non-physiological nature of the 2KQ mutant by citing published evidence that TDP-43 acetylation occurs in ALS patient spinal cord and is upregulated under oxidative and proteotoxic stress conditions. While these references are real and support the relevance of acetylation as a pathological post-translational modification, they do not resolve the central concern: there is no quantification of how much endogenous TDP-43 is acetylated at the specific lysine residues mimicked by 2KQ in degenerating human neurons, and no evidence that the degree of RNA-binding disruption imposed by the double glutamine substitution is ever achieved by endogenous acetylation in vivo. The 2KQ mutant eliminates RNA binding essentially completely, whereas physiological acetylation events are graded, reversible, and likely partial. The response conflates the existence of TDP-43 acetylation as a phenomenon with validation that 2KQ is a physiologically accurate model of that phenomenon. None of the new experiments address the request to test whether wild-type TDP-43 expressed at near-physiological levels, or a bona fide heterozygous ALS-linked TARDBP mutant in iPSC-derived neurons, responds to XPO1 modulation in a qualitatively similar fashion. Until this is shown, the mechanistic conclusions of this paper remain constrained to a highly artificial overexpression system and cannot be extrapolated to physiological or pathological TDP-43 biology with confidence.

(2) The homozygous K181E organoid model is still not adequately justified, and no heterozygous comparison has been provided.

The authors acknowledge that the homozygous background is "more sensitive for detecting phospho-TDP-43" and argue that homozygous conditions are commonly used in experimental TDP-43 research. However, the critical issue is not whether homozygous models are used in general, but whether the homozygous background specifically alters the relative contribution of cytoplasmic aggregation versus nuclear RNA-processing dysfunction in this study. In a homozygous K181E model, both alleles produce an RNA-binding-defective TDP-43, meaning that every molecule of endogenous TDP-43 in the cell is dysfunctional. This is categorically different from the patient situation in which one wild-type allele is present, and it may substantially exaggerate nuclear loss-of-function relative to cytoplasmic gain-of-function phenotypes. The authors have not performed the requested comparison with heterozygous K181E/+ organoids, nor have they acknowledged that the organoid genotype itself could bias the interpretation of what KPT-276 treatment rescues. Given that the organoid section is now the sole in-disease-model validation of the XPO1 mechanism, this limitation is more consequential than it was in the original submission.

(3) The new semi-permeabilized cell data is a genuine contribution, but the mechanistic interpretation remains insufficiently constrained.

The development of the streptolysin O semi-permeabilized cell reconstitution system is the most substantive new addition to this revision. The finding that LMB-stabilized anisosomes resist cytosol washout but dissolve upon RNase T1 treatment is interesting and provides a plausible indirect mechanism: XPO1 inhibition retains nuclear RNA, and this elevated nuclear RNA availability contributes to maintaining the liquid LLPS state of the TDP-43 2KQ condensate. This is a meaningful mechanistic advance and deserves credit. However, several important limitations of this new data are not adequately discussed. First, RNase T1 degrades single-stranded RNA globally during permeabilization, so the experiment does not identify which specific RNA species stabilize the anisosome, nor whether these are pre-mRNA splicing intermediates, mature mRNA, non-coding RNA, or another class. Second, the same nuclear export blockade that retains RNA will also retain the nuclear concentrations of many RNA-binding proteins, splicing factors, and other XPO1-dependent cargos. The RNase T1 experiment does not exclude the possibility that the relevant effect is mediated by an RNA-binding protein whose nuclear concentration increases upon LMB treatment and which, upon RNase digestion, can no longer engage TDP-43 or the anisosome shell. Third, the permeabilized cell system is by definition not intact and has lost cytosolic factors; whether the RNA-dependent stabilization of anisosomes operates in the same way in intact cells during physiological or pathological nuclear export perturbation is an assumption, not a demonstrated fact. The authors should more carefully frame these data as hypothesis-generating and explicitly note these alternative interpretations in the Discussion.

(4) The conceptual asymmetry between XPO1 inhibition and XPO1 overexpression phenotypes is not resolved by the new mechanism.

The paper continues to present two XPO1 perturbation phenotypes that are difficult to reconcile within a single mechanistic model. XPO1 inhibition enlarges anisosomes, maintains their liquid character by FRAP, and retains them in the nucleus. XPO1 overexpression also enlarges TDP-43 puncta, but these are FRAP-impaired, gel-like, and appear in the cytoplasm. The RNA-retention model proposed by the new semi-permeabilized data explains why XPO1 inhibition stabilizes the liquid state, but it does not explain why XPO1 overexpression drives the opposite outcome: gel-like hardening and cytoplasmic redistribution. If increased nuclear RNA availability is the key variable downstream of XPO1 inhibition, then XPO1 overexpression would be expected to decrease nuclear RNA and thereby destabilize anisosomes toward dissolution or hardening. The paper does not test whether nuclear RNA levels are indeed altered by XPO1 overexpression, nor whether the cytoplasmic gel-like puncta seen in XPO1-overexpressing cells are RNA-poor relative to control anisosomes. The revised Discussion does not engage with this asymmetry in a satisfying way, and the figure model remains qualitative. A quantitative or at least semi-quantitative model that accounts for both arms of the XPO1 perturbation is needed.

(5) The removal of RNA-seq data weakens rather than strengthens the organoid section.

The authors have removed the bulk RNA-seq analysis from the revised manuscript in response to concerns that the modest transcriptional rescue was being over-interpreted. While the decision to remove over-interpretation is appropriate, the result is that the organoid section now rests entirely on pTDP-43 immunostaining as its sole readout. The revised paper thus uses reduction in immunofluorescent pTDP-43 puncta in homozygous K181E organoids as the only evidence that nuclear export inhibition mitigates TDP-43 proteinopathy in a disease-relevant context. This is a weaker evidentiary base than before the revision, not an improvement. The originally requested more sensitive orthogonal readouts, including biochemical fractionation for SDS-insoluble TDP-43, filter-trap assays, or RNA aptamer-based detection of TDP-43 aggregates, remain absent. Without at least one additional independent measure confirming that cytoplasmic TDP-43 aggregation is genuinely reduced rather than simply rendered antigenically undetectable, the organoid conclusion is not adequately supported. At minimum, the authors should provide total and cytoplasmic TDP-43 fractionation data from organoid lysates to corroborate the immunostaining result.

(6) No functional neuronal readout has been provided for the organoid model.

The organoid section now makes the claim that "nuclear export is required for the formation of p-TDP-43-containing aggregates in a disease-relevant organoid model," but no measure of neuronal health, integrity, or function is reported in association with this. Even a simple assessment of neuron survival by TUJ1 or MAP2 quantification, neurite complexity, or cleaved caspase-3 staining before and after KPT-276 treatment would substantially strengthen the biological significance of the pTDP-43 reduction. The current data establish a pharmacological effect on a pathological marker but do not demonstrate that this has any consequence for neuronal biology in the organoid, which is what the disease-relevance framing implies.

(7) The abstract and title continue to overstate the mechanistic conclusions.

Despite the stated intent to reframe the study as a screening study and to temper the conclusions, the revised abstract retains the language: "These findings establish nuclear export as a key regulator of TDP-43 phase transitions and define a mechanistic framework that links altered nuclear transport and phase dynamics to TDP-43 aggregation potential." Similarly, the Discussion still states: "a particularly compelling aspect of our study is the discovery that the nuclear export receptor XPO1 governs TDP-43 liquid-to-solid transitions and subcellular localization." The word "governs" and the phrase "establish nuclear export as a key regulator" are not warranted by data that derive entirely from an overexpressed acetylation-mimetic mutant in a colon cancer cell line and a homozygous K181E organoid model. A more accurate framing would describe these findings as identifying nuclear export as one of several cellular processes that modulate TDP-43 phase behavior in a sensitized model system, with an indirect RNA-mediated mechanism that remains to be defined at the molecular level. The title change from "governs" to "modulates" is appreciated but does not extend into the abstract and Discussion, where the strong causal language persists.

(8) Individual siRNA knockdown validation for XPO1 has not been provided.

The authors argue that validation with 6 independent siRNAs across two rounds of screening, combined with convergent pharmacological data, is sufficient to establish XPO1 as a genuine hit. While the convergence of chemical and genetic evidence is reassuring, the specific request was for protein-level confirmation of XPO1 knockdown efficiency in the DLD1 TDP-43 2KQ cells used for mechanistic follow-up, together with demonstration that the anisosome phenotype is specifically caused by loss of XPO1 and not by off-target effects. This is a straightforward experiment, and its absence is particularly notable given that the entire mechanistic XPO1 narrative hinges on this specificity. At minimum, an immunoblot confirming XPO1 protein depletion in cells treated with the siRNA pool identified in the screen, in the same cell background and induction conditions as the follow-up experiments, should be provided.

(9) The identity of XPO1-dependent cargos that regulate anisosome dynamics remains entirely unknown.

The authors acknowledge that XPO1 does not directly bind TDP-43 and that the mechanism is likely indirect. The new RNA data provides one plausible indirect pathway. However, the possibility that one or more specific RNA-binding proteins or splicing factors, whose nuclear levels rise upon XPO1 inhibition, are the proximate drivers of anisosome stabilization has not been addressed. This matters because if the relevant mechanism operates through a specific cargo rather than bulk RNA retention, the model for how nuclear export connects to TDP-43 aggregation in disease would be fundamentally different. The authors decline to pursue adaptor identification on grounds of scope, which is a defensible position for future work. However, the framing should explicitly state that the current data cannot distinguish between bulk RNA retention and cargo-specific effects, and that the conclusion that nuclear export modulates TDP-43 phase behavior via RNA accumulation is a working hypothesis supported by but not proven by the RNase T1 experiment.

Minor remaining issues.

The number of independent iPSC clones and organoid batches used for the KPT-276 treatment experiment is now stated as two batches per condition, which is minimal for a 3D organoid study and does not fully address the concern about clone-level variability. Ideally, organoids from at least two independently derived isogenic clones per genotype would be used. The mCherry overexpression control added in Supplemental Figure 4 is a useful addition and is acknowledged. The immunoblotting confirmation that drug treatments do not alter total TDP-43 levels addresses a prior concern adequately. The addition of the sentence noting that anisosomes have not been validated in human patient samples is appreciated and appropriate. Statistical detail has been improved in figure legends. These minor improvements are noted positively but do not compensate for the major unresolved concerns above.

# Referee report: Reviewer #2 (Public review)

**DOI:** 10.7554/eLife.110172.4.sa2

This manuscript addresses an important and timely question in TDP-43 biology by systematically identifying regulators of TDP-43 anisosome formation, with a particular focus on nuclear export via XPO1. Using a combination of unbiased chemical screening, genetic perturbation, and advanced imaging approaches, the authors propose that inhibition of nuclear export modulates the abundance and biophysical properties of TDP-43 anisosomes. They further strengthen their findings by introducing an additional model system, a semi-permeabilized in vitro assay, which provides mechanistic evidence that XPO1 activity prevents anisosome dissolution by retaining nuclear RNAs. The study is conceptually innovative and has potential relevance for neurodegenerative diseases characterized by TDP-43 pathology. Some minor concerns remain, mostly about experimental design of the newly added data.

Strengths:

(1) The study employs an unbiased, hypothesis-free compound screen to identify regulators of TDP-43 anisosome formation, which is a major strength and reduces confirmation bias.

(2) The authors combine chemical and genetic screening approaches, providing orthogonal validation of key pathways and increasing confidence in the biological relevance of top hits.

(3) The focus on biophysical properties of TDP-43 assemblies, assessed through imaging and FRAP, moves beyond simple presence/absence of aggregates and provides mechanistic insight into the biophysical states of TDP-43.

(4) The use of multiple experimental modalities, including live-cell imaging, FRAP, pharmacological perturbation, and transcriptomic analysis, reflects a technically sophisticated and ambitious study design.

(5) The authors attempt to extend findings beyond immortalized cancer cell lines by incorporating organoid models, demonstrating awareness of disease relevance and translational importance.

(6) The authors extend their study by incorporating a semi-permeabilized in vitro system, which provides compelling evidence that inhibition of nuclear export promotes the retention of nuclear anisosomes, an effect driven by the accumulation of nuclear RNAs.

Overall, the manuscript is clearly written and logically structured, making complex experimental workflows accessible and the central hypotheses easy to follow.

Weaknesses:

(1) The manuscript has significantly improved with the revisions. Some experimental procedures and method details, as well has statements remain incompletely described:

a) What is the smear in Figure S1 after VLX treatment?

b) The authors state that "The reduction in TDP-43 signal was not due to protein elimination.", however no data is provided to support that statement.

c) The authors state that "TDP-43 shifts from phase-separated state to a soluble state ...", however no data is provided to support that statement.

d) Why did the authors choose cow lover cytosol for this study?

e) The experimental setup for supplementing with cytosol/ATP/GTP is unclear. A more detailed schematic would be helpful to understand at what stage in the experiment these factors were added. Which step of the protocol was performed at 37 {degree sign}C, which is indicated in the figure schematic but not described in the methods.

f) In the organoid model, the authors mention that they observe similar levels of total TDP-43, however they do not provide quantification. Instead, they provide a graph that shows highly significant changes in nuclear TDP-43, which was not addressed in the text.

Additionally, some questions remain unclear:

(1) The anisosomes induced by ATP/GTP or cytosol are insufficiently characterized. It remains unclear whether these structures correspond to canonical ring-shaped anisosomes, and whether they exhibit dynamic (liquid-like) or more static (gel-like) properties.

(2) The contribution of the cytosol and ATP/GTP supplementation experiments to the overall narrative is unclear. While the findings are intriguing, their interpretation within the context of the study is not well articulated. In particular, the rationale for including cytosol is not sufficiently justified, given that ATP/GTP alone induces a pronounced effect, whereas cytosol alone does not.

(3) The authors should address why endogenous XPO1 does not co-localize with anisosomes, whereas overexpressed XPO1 does. This raises the possibility that the observed co-localization may be an artifact of non-physiological protein levels, which should be discussed.

(4) The iPSC-based model remains insufficiently characterized. While the authors propose that this system recapitulates the accumulation of liquid and solid aggregates resembling anisosomes, it is unclear whether this phenotype is robustly observed and whether KPT treatment effectively modulates it.

(5) The rationale for the selected treatment durations is unclear, and the timing appears inconsistent across experiments (ranging from 3 to 16 hours), including within experiments involving the same compound. This variability should be justified or standardized.

(6) Several figure legends require clarification:

a) In the section stating "Collectively, our results suggest that the stability and dynamics of anisosomes are modulated by XPO1-mediated nuclear export ...", the cited figure appears to be incorrect. This should refer to Figure 5L rather than Figure 5J.

b) Figure 1B: Please specify the number of replicates per concentration, the number of cells analyzed, and the model used for regression analysis. Additionally, the legend indicates a treatment duration of 15 hours, whereas Figure 1A states 24 hours.

c) Figure 2G: The authors state "7 anisosomes per condition," but the graph displays only 4-6 data points. Please clarify what each data point represents.

d) Figures 3B and 3G: Please clarify whether a defined threshold was used to determine a "reduction in anisosome number."

e) Figure 4B: These do not represent biological replicates, as all samples derive from a single cell line; rather, they constitute independent experimental replicates.

f) Figures 5B and 5H: The legend states "n = 3 biological repeats," but the number of data points shown appears higher. Please clarify.

g) Figures 5K, 6C, and 6E: "Mean Fluorescence Intensity (MPI)" should be corrected to "MFI."

h) Figure 6C: Please include the number of cells analyzed and provide relevant statistical measures (e.g., R², p-value).

i) Figure 6D: The experimental timeline is unclear. Please specify the duration of incubation and the timing of each step.

j) Figure 7B: Improved labeling is needed (e.g., clarification of "mean spot volume") to better align with the figure legend.

# Referee report: Reviewer #3 (Public review)

**DOI:** 10.7554/eLife.110172.4.sa3

Summary:

TDP-43 proteinopathy is broadly found in neurodegenerative diseases. This manuscript investigates how nuclear export influences the biophysical properties of TDP-43. The authors use a combination of chemical screening and genome-wide siRNA screening to identify pathways that modulate TDP-43 liquid-to-solid transitions. Overall, the study employs a broad array of approaches and addresses an important question in TDP-43 pathobiology. The identification of nuclear export as a central regulator is compelling and conceptually aligns with the emerging view that TDP-43 nucleocytoplasmic trafficking is a major defect in neurodegeneration.

Strengths:

This work integrates chemical and genetic screening to identify novel modifiers. The candidates were validated in both reporter cell lines and iPS-differentiated organoids. The findings support the nucleocytoplasmic transport is important for the biophysical properties of TDP-43.

Comments on revised version.

The manuscript has been improved with more data and clarification. The RNase T1 treatment experiment suggests that RNA is required for anisosome integrity. However, this does not directly demonstrate LMB increases nuclear RNA availability as changes in protein composition or other RNA-dependent mechanisms may also contribute. The conclusion and discussion need to be edited to consider these alternative scenarios. Overall, as most of the evidence remains indirect, the manuscript should avoid overinterpretation regarding the mechanisms underlying TDP-43 phase transition and aggregation.

# Author comment: Author response

**DOI:** 10.7554/eLife.110172.4.sa4

**Authors:** Natalie Chin, Qi Zhang, Jizhong Zou, Ken Chih-Chien Cheng, Wei Zheng, Yihong Ye

**Affiliations:** National Institute of Diabetes and Digestive and Kidney Diseases Bethesda United States; National Center for Advancing Translational Sciences BETHESDA United States; NHLBI/NIH Bethesda United States; National Institutes of Health Rockville United States; National Center for Advancing Translational Sciences Rockville United States; National Institute of Diabetes and Digestive and Kidney Diseases Bethesda United States

The following is the authors’ response to the previous reviews

**Public Reviews:**

**Reviewer #1 (Public review):**

This revised manuscript represents a partial response to the concerns raised in the first round of review. The authors have made one genuine mechanistic addition in the form of the semi-permeabilized cell reconstitution assay, removed the most overreaching conclusions regarding the contribution of cytoplasmic TDP-43 aggregation to disease, and made several minor presentational improvements. However, the central weaknesses of the original submission remain substantially unaddressed. The exclusive reliance on non-physiological TDP-43 variants, the incompletely resolved mechanism linking XPO1 to TDP-43 phase behavior, and the limited organoid validation continue to limit confidence in the major claims. The authors have, in several instances, responded by removing contested data rather than by providing the additional evidence that was requested.

(1) The justification for the 2KQ acetylation-mimetic system remains inadequate.

The authors respond to the concern about the non-physiological nature of the 2KQ mutant by citing published evidence that TDP-43 acetylation occurs in ALS patient spinal cord and is upregulated under oxidative and proteotoxic stress conditions. While these references are real and support the relevance of acetylation as a pathological post-translational modification, they do not resolve the central concern: there is no quantification of how much endogenous TDP-43 is acetylated at the specific lysine residues mimicked by 2KQ in degenerating human neurons, and no evidence that the degree of RNA-binding disruption imposed by the double glutamine substitution is ever achieved by endogenous acetylation in vivo. The 2KQ mutant eliminates RNA binding essentially completely, whereas physiological acetylation events are graded, reversible, and likely partial. The response conflates the existence of TDP-43 acetylation as a phenomenon with validation that 2KQ is a physiologically accurate model of that phenomenon. None of the new experiments address the request to test whether wild-type TDP-43 expressed at near-physiological levels, or a bona fide heterozygous ALS-linked TARDBP mutant in iPSC-derived neurons, responds to XPO1 modulation in a qualitatively similar fashion. Until this is shown, the mechanistic conclusions of this paper remain constrained to a highly artificial overexpression system and cannot be extrapolated to physiological or pathological TDP-43 biology with confidence.

We agree with the reviewer that the TDP-43 2KQ mutant is a non-physiological variant. To address this concern, we have removed all statements that extrapolate our findings to disease pathogenesis. As reflected in the revised title, we now present this study as an investigation of factors that modulate TDP-43 phase transition and aggregation using a sensitized model system, rather than as a direct disease model. The Abstract and Discussion have also been revised accordingly.

The choice of the 2KQ mutant was dictated by the requirements of the screening strategy. To identify modulators of TDP-43 phase transition, it was necessary to use a TDP-43 variant that reliably undergoes phase separation in cells within an experimentally practical timeframe. In this context, we believe the 2KQ mutant provides a suitable and justified experimental tool. We have revised the manuscript to clearly distinguish observations made with this engineered construct from conclusions regarding physiological or disease-associated TDP-43.

(2) The homozygous K181E organoid model is still not adequately justified, and no heterozygous comparison has been provided.

The authors acknowledge that the homozygous background is "more sensitive for detecting phospho-TDP-43" and argue that homozygous conditions are commonly used in experimental TDP-43 research. However, the critical issue is not whether homozygous models are used in general, but whether the homozygous background specifically alters the relative contribution of cytoplasmic aggregation versus nuclear RNA-processing dysfunction in this study. In a homozygous K181E model, both alleles produce an RNA-binding-defective TDP-43, meaning that every molecule of endogenous TDP-43 in the cell is dysfunctional. This is categorically different from the patient situation in which one wild-type allele is present, and it may substantially exaggerate nuclear loss-of-function relative to cytoplasmic gain-of-function phenotypes. The authors have not performed the requested comparison with heterozygous K181E/+ organoids, nor have they acknowledged that the organoid genotype itself could bias the interpretation of what KPT-276 treatment rescues. Given that the organoid section is now the sole in-disease-model validation of the XPO1 mechanism, this limitation is more consequential than it was in the original submission.

We agree with the reviewer and have removed all speculative statements regarding the relative contributions of cytoplasmic aggregation and RNA splicing defects to disease pathogenesis. The organoid section has also been revised to focus solely on the experimental findings. Specifically, we only present evidence that inhibition of nuclear export in a sensitized organoid model promotes the accumulation of cytoplasmic phosphorylated TDP-43 without making broader claims regarding its role in disease pathogenesis.

(3) The new semi-permeabilized cell data is a genuine contribution, but the mechanistic interpretation remains insufficiently constrained.

The development of the streptolysin O semi-permeabilized cell reconstitution system is the most substantive new addition to this revision. The finding that LMB-stabilized anisosomes resist cytosol washout but dissolve upon RNase T1 treatment is interesting and provides a plausible indirect mechanism: XPO1 inhibition retains nuclear RNA, and this elevated nuclear RNA availability contributes to maintaining the liquid LLPS state of the TDP-43 2KQ condensate. This is a meaningful mechanistic advance and deserves credit. However, several important limitations of this new data are not adequately discussed. First, RNase T1 degrades single-stranded RNA globally during permeabilization, so the experiment does not identify which specific RNA species stabilize the anisosome, nor whether these are pre-mRNA splicing intermediates, mature mRNA, non-coding RNA, or another class. Second, the same nuclear export blockade that retains RNA will also retain the nuclear concentrations of many RNA-binding proteins, splicing factors, and other XPO1-dependent cargos. The RNase T1 experiment does not exclude the possibility that the relevant effect is mediated by an RNA-binding protein whose nuclear concentration increases upon LMB treatment and which, upon RNase digestion, can no longer engage TDP-43 or the anisosome shell. Third, the permeabilized cell system is by definition not intact and has lost cytosolic factors; whether the RNA-dependent stabilization of anisosomes operates in the same way in intact cells during physiological or pathological nuclear export perturbation is an assumption, not a demonstrated fact. The authors should more carefully frame these data as hypothesis-generating and explicitly note these alternative interpretations in the Discussion.

We have now added some sentences on page 11 to acknowledge the limitation of our experiments. It reads as “However, our data does not exclude other RNA species or RNA-binding proteins as anisosome stabilizer. Whether RNA-dependent stabilization of anisosomes operates in the same way in intact cells also requires further validation.”

(4) The conceptual asymmetry between XPO1 inhibition and XPO1 overexpression phenotypes is not resolved by the new mechanism.

The paper continues to present two XPO1 perturbation phenotypes that are difficult to reconcile within a single mechanistic model. XPO1 inhibition enlarges anisosomes, maintains their liquid character by FRAP, and retains them in the nucleus. XPO1 overexpression also enlarges TDP-43 puncta, but these are FRAP-impaired, gel-like, and appear in the cytoplasm. The RNA-retention model proposed by the new semi-permeabilized data explains why XPO1 inhibition stabilizes the liquid state, but it does not explain why XPO1 overexpression drives the opposite outcome: gel-like hardening and cytoplasmic redistribution. If increased nuclear RNA availability is the key variable downstream of XPO1 inhibition, then XPO1 overexpression would be expected to decrease nuclear RNA and thereby destabilize anisosomes toward dissolution or hardening. The paper does not test whether nuclear RNA levels are indeed altered by XPO1 overexpression, nor whether the cytoplasmic gel-like puncta seen in XPO1-overexpressing cells are RNA-poor relative to control anisosomes. The revised Discussion does not engage with this asymmetry in a satisfying way, and the figure model remains qualitative. A quantitative or at least semi-quantitative model that accounts for both arms of the XPO1 perturbation is needed.

We thank the reviewer for this point. We have now explicitly mentioned in the discussion that the effect of XPO-1 on anisosome dynamics is likely mediated by an indirect mechanism. We also acknowledge that we do not fully understand why over-expression of XPO-1 causes TDP-43 to accumulate in gel-like structures in the cytoplasm. Although we did not check whether overexpression of XPO1 increases cargo export, we cited studies showing that over-expressed XPO1 disrupts the normal distribution of cargos between nucleus and cytoplasm on page 7. To avoid confusion, we also revised the result part on page 7, emphasizing on the difference rather than the similar increase in puncta size by opposing manipulations.

(5) The removal of RNA-seq data weakens rather than strengthens the organoid section.

The authors have removed the bulk RNA-seq analysis from the revised manuscript in response to concerns that the modest transcriptional rescue was being over-interpreted. While the decision to remove over-interpretation is appropriate, the result is that the organoid section now rests entirely on pTDP-43 immunostaining as its sole readout. The revised paper thus uses reduction in immunofluorescent pTDP-43 puncta in homozygous K181E organoids as the only evidence that nuclear export inhibition mitigates TDP-43 proteinopathy in a disease-relevant context. This is a weaker evidentiary base than before the revision, not an improvement. The originally requested more sensitive orthogonal readouts, including biochemical fractionation for SDS-insoluble TDP-43, filter-trap assays, or RNA aptamer-based detection of TDP-43 aggregates, remain absent. Without at least one additional independent measure confirming that cytoplasmic TDP-43 aggregation is genuinely reduced rather than simply rendered antigenically undetectable, the organoid conclusion is not adequately supported. At minimum, the authors should provide total and cytoplasmic TDP-43 fractionation data from organoid lysates to corroborate the immunostaining result.

We removed the RNA-seq analysis because both the reviewers and editors agreed that the modest transcriptional rescue should not be overinterpreted. Upon reconsideration, we believe that reinstating these data would not address the reviewer's principal concern, namely whether nuclear export inhibition reduces TDP-43 aggregation in organoids. We have therefore chosen to limit our conclusions to the direct observation supported by the current data, namely a reduction in cytoplasmic phosphorylated TDP-43 immunoreactivity. We also add a sentence to acknowledge that “Whether the reduction in pTDP-43 immunoreactivity reflects a decrease in insoluble TDP-43 aggregates remains to be determined” on page 10.

(6) No functional neuronal readout has been provided for the organoid model.

The organoid section now makes the claim that "nuclear export is required for the formation of p-TDP-43-containing aggregates in a disease-relevant organoid model," but no measure of neuronal health, integrity, or function is reported in association with this. Even a simple assessment of neuron survival by TUJ1 or MAP2 quantification, neurite complexity, or cleaved caspase-3 staining before and after KPT-276 treatment would substantially strengthen the biological significance of the pTDP-43 reduction. The current data establish a pharmacological effect on a pathological marker but do not demonstrate that this has any consequence for neuronal biology in the organoid, which is what the disease-relevance framing implies.

We thank the reviewer for this helpful suggestion. We agree that assessments of neuronal survival or function would be important if the manuscript were claiming that nuclear export inhibition improves neuronal health or rescues disease phenotypes in the organoid model. However, in response to the reviewers' comments regarding the physiological relevance of the homozygous K181E organoids, we have substantially revised both the framing and interpretation of this section.

Specifically, we have revised the statement to read, "These results imply that maintaining TDP-43 in the nuclear demixed liquid state might diminish p-TDP-43 accumulation but whether the reduction of pTDP-43 immunoreactivity reflects a decrease in insoluble TDP-43 aggregates remains to be determined," thereby limiting our conclusion to the direct experimental observation. We no longer make claims regarding disease modification or functional rescue in the organoid model. Given this revised scope, we believe that additional measurements of neuronal survival or function, while certainly of interest, are not essential to support the conclusions presented in this study. We have also revised the conclusion to explicitly acknowledge that the functional consequences of reducing p-TDP-43-positive puncta (whether this can be translated to reduced aggregation) remain to be determined by future studies (page 10).

(7) The abstract and title continue to overstate the mechanistic conclusions.

Despite the stated intent to reframe the study as a screening study and to temper the conclusions, the revised abstract retains the language: "These findings establish nuclear export as a key regulator of TDP-43 phase transitions and define a mechanistic framework that links altered nuclear transport and phase dynamics to TDP-43 aggregation potential." Similarly, the Discussion still states: "a particularly compelling aspect of our study is the discovery that the nuclear export receptor XPO1 governs TDP-43 liquid-to-solid transitions and subcellular localization." The word "governs" and the phrase "establish nuclear export as a key regulator" are not warranted by data that derive entirely from an overexpressed acetylation-mimetic mutant in a colon cancer cell line and a homozygous K181E organoid model. A more accurate framing would describe these findings as identifying nuclear export as one of several cellular processes that modulate TDP-43 phase behavior in a sensitized model system, with an indirect RNA-mediated mechanism that remains to be defined at the molecular level. The title change from "governs" to "modulates" is appreciated but does not extend into the abstract and Discussion, where the strong causal language persists.

We have revised the title of the paper, reframing it as a screen that reveals modulators of TDP-43 phase separation. The last sentence of the abstract is also revised accordingly. It now reads as “These findings identify multiple modulators of TDP-43 phase transitions in a sensitized model system and establish a framework for further dissecting the link between nuclear transport and TDP-43 phase dynamics.” We also tone down our conclusions and discussions.

(8) Individual siRNA knockdown validation for XPO1 has not been provided.

The authors argue that validation with 6 independent siRNAs across two rounds of screening, combined with convergent pharmacological data, is sufficient to establish XPO1 as a genuine hit. While the convergence of chemical and genetic evidence is reassuring, the specific request was for protein-level confirmation of XPO1 knockdown efficiency in the DLD1 TDP-43 2KQ cells used for mechanistic follow-up, together with demonstration that the anisosome phenotype is specifically caused by loss of XPO1 and not by off-target effects. This is a straightforward experiment, and its absence is particularly notable given that the entire mechanistic XPO1 narrative hinges on this specificity. At minimum, an immunoblot confirming XPO1 protein depletion in cells treated with the siRNA pool identified in the screen, in the same cell background and induction conditions as the follow-up experiments, should be provided.

While we agree with the reviewer that studies relying on siRNA should provide sufficient information regarding knockdown efficiency and specificity, we respectfully disagree that this should be a major concern in the present study. As explained in the manuscript, we deliberately chose not to pursue mechanistic studies using chronic XPO1 knockdown because prolonged depletion of this essential nuclear export factor is likely to produce secondary effects that could complicate data interpretation. Instead, we employed multiple chemically distinct XPO1 inhibitors to achieve acute inhibition, thereby minimizing indirect consequences while providing a more appropriate approach for mechanistic analysis.

We agree that assessing knockdown efficiency is technically straightforward. However, because our mechanistic conclusions are based primarily on acute pharmacological inhibition rather than siRNA-mediated depletion, we prioritized experiments that directly addressed the central mechanistic questions raised by the reviewers, particularly the semi-permeabilized cell assay. Moreover, the XPO1 inhibitors used in this study are well-characterized, highly specific compounds that have been extensively validated and widely used in the literature. We therefore believe that our experimental strategy provides a reliable basis for the conclusions presented.

(9) The identity of XPO1-dependent cargos that regulate anisosome dynamics remains entirely unknown.

The authors acknowledge that XPO1 does not directly bind TDP-43 and that the mechanism is likely indirect. The new RNA data provides one plausible indirect pathway. However, the possibility that one or more specific RNA-binding proteins or splicing factors, whose nuclear levels rise upon XPO1 inhibition, are the proximate drivers of anisosome stabilization has not been addressed. This matters because if the relevant mechanism operates through a specific cargo rather than bulk RNA retention, the model for how nuclear export connects to TDP-43 aggregation in disease would be fundamentally different. The authors decline to pursue adaptor identification on grounds of scope, which is a defensible position for future work. However, the framing should explicitly state that the current data cannot distinguish between bulk RNA retention and cargo-specific effects, and that the conclusion that nuclear export modulates TDP-43 phase behavior via RNA accumulation is a working hypothesis supported by but not proven by the RNase T1 experiment.

We thank the reviewer for this helpful suggestion. We have now added a sentence on page 11, which state that “our data does not exclude other RNA species or RNA-binding proteins as anisosome stabilizer. Whether RNA-dependent stabilization of anisosomes operates in the same way in intact cells also requires validation.”

Minor remaining issues.

The number of independent iPSC clones and organoid batches used for the KPT-276 treatment experiment is now stated as two batches per condition, which is minimal for a 3D organoid study and does not fully address the concern about clone-level variability. Ideally, organoids from at least two independently derived isogenic clones per genotype would be used. The mCherry overexpression control added in Supplemental Figure 4 is a useful addition and is acknowledged. The immunoblotting confirmation that drug treatments do not alter total TDP-43 levels addresses a prior concern adequately. The addition of the sentence noting that anisosomes have not been validated in human patient samples is appreciated and appropriate. Statistical detail has been improved in figure legends. These minor improvements are noted positively but do not compensate for the major unresolved concerns above.

We thank the reviewer for his/her appreciation of our previous revision. We hope that the new changes now satisfactorily address the remaining concerns.

**Reviewer #2 (Public review):**

This manuscript addresses an important and timely question in TDP-43 biology by systematically identifying regulators of TDP-43 anisosome formation, with a particular focus on nuclear export via XPO1. Using a combination of unbiased chemical screening, genetic perturbation, and advanced imaging approaches, the authors propose that inhibition of nuclear export modulates the abundance and biophysical properties of TDP-43 anisosomes. They further strengthen their findings by introducing an additional model system, a semi-permeabilized in vitro assay, which provides mechanistic evidence that XPO1 activity prevents anisosome dissolution by retaining nuclear RNAs. The study is conceptually innovative and has potential relevance for neurodegenerative diseases characterized by TDP-43 pathology. Some minor concerns remain, mostly about experimental design of the newly added data.

Strengths:

(1) The study employs an unbiased, hypothesis-free compound screen to identify regulators of TDP-43 anisosome formation, which is a major strength and reduces confirmation bias.

(2) The authors combine chemical and genetic screening approaches, providing orthogonal validation of key pathways and increasing confidence in the biological relevance of top hits.

(3) The focus on biophysical properties of TDP-43 assemblies, assessed through imaging and FRAP, moves beyond simple presence/absence of aggregates and provides mechanistic insight into the biophysical states of TDP-43.

(4) The use of multiple experimental modalities, including live-cell imaging, FRAP, pharmacological perturbation, and transcriptomic analysis, reflects a technically sophisticated and ambitious study design.

(5) The authors attempt to extend findings beyond immortalized cancer cell lines by incorporating organoid models, demonstrating awareness of disease relevance and translational importance.

(6) The authors extend their study by incorporating a semi-permeabilized in vitro system, which provides compelling evidence that inhibition of nuclear export promotes the retention of nuclear anisosomes, an effect driven by the accumulation of nuclear RNAs.

Overall, the manuscript is clearly written and logically structured, making complex experimental workflows accessible and the central hypotheses easy to follow.

We thank the reviewer for acknowledging the strength and the potential significance of our study.

Weaknesses:

(1) The manuscript has significantly improved with the revisions. Some experimental procedures and method details, as well has statements remain incompletely described:

(a) What is the smear in Figure S1 after VLX treatment?

We thank the reviewers for the positive assessment. We do not know why VLX treatment causes a fraction of TDP-43 to migrate slowly. We suspect that it may form detergent-insoluble aggregates. However, we cannot be sure whether this occurred during drug treatment or sample preparation. We now add a sentence in the figure legend to clarify this point.

(b) The authors state that "The reduction in TDP-43 signal was not due to protein elimination.", however no data is provided to support that statement.

We reasoned that the reduction in TDP-43 was probably not caused by protein elimination because the puncta could be reformed when permeabilized cells were incubated with exogenously added cytosol and ATP/GTP. We have revised the text to avoid this confusion. The revision on page 8 reads as “The reduction in TDP-43 signal probably resulted from a shift of TDP-43 from a phase-separated high fluorescent state into a soluble state with reduced fluorescence intensity (Zhang et al., 2026). We attributed this phenotype to the depletion of cytosolic factors and ATP during cell permeabilization because it is known that anisosome formation and maintenance require HSP70, a cytosolic ATPase (Yu et al., 2021).”.

(c) The authors state that "TDP-43 shifts from phase-separated state to a soluble state ...", however no data is provided to support that statement.

Since TDP-43 protein was apparently still in the nucleus after cell permeabilization (see above) but became invisible, the best interpretation is that the protein is shifted into a soluble state, which reduces the fluorescence intensity substantially. We have revised the text to clarify this point. We also cited a recent study showing that EGFP-alpha-synuclein oligomerization/aggregation enhances its fluorescence intensity in cells.

(d) Why did the authors choose cow lover cytosol for this study?

The main reason is because we have access to a large amount of cow liver cytosol that is known to have activities in in vitro reconstitution assays. We now cite several papers from us that reported the use of the same cytosol in other in vitro assays in the method (page 14).

(e) The experimental setup for supplementing with cytosol/ATP/GTP is unclear. A more detailed schematic would be helpful to understand at what stage in the experiment these factors were added. Which step of the protocol was performed at 37 {degree sign}C, which is indicated in the figure schematic but not described in the methods.

We now revise the schematic in Figure 6A and include more details in the method and figure legend.

(f) In the organoid model, the authors mention that they observe similar levels of total TDP-43, however they do not provide quantification. Instead, they provide a graph that shows highly significant changes in nuclear TDP-43, which was not addressed in the text.

The total TDP-43 level was shown by immunostaining in green in Figure 7. This was used as a control to show that the increase in p-TDP-43 was not simply caused by an overall increase in its protein level. We have added the quantification to Figure 7C. We also discuss the reduced nuclear TDP-43 in organoids bearing the disease mutation in the main text.

Additionally, some questions remain unclear:

(1) The anisosomes induced by ATP/GTP or cytosol are insufficiently characterized. It remains unclear whether these structures correspond to canonical ring-shaped anisosomes, and whether they exhibit dynamic (liquid-like) or more static (gel-like) properties.

We agree that the structures reformed after incubating permeabilized cells with cytosol and ATP/GTP are not fully characterized. Due to their small size, we could not see the typical ring-shaped anisosome morphology. FRAP experiment is also tricky. Due to these issues, we have revised the text to acknowledge that we do not know the exact identity of these structures. We speculate that they are anisosome-related because like anisosome formation, it depends on cytosolic factor and energy (page 9). It is worth noting that whether these structures are anisosomes is not the main conclusion of this experiment. We conclude from this experiment that TDP-43 was still in the nucleus after cell permeabilization (not degraded). The fact that we could not see the protein likely because the protein was in a low-fluorescence soluble state.

(2) The contribution of the cytosol and ATP/GTP supplementation experiments to the overall narrative is unclear. While the findings are intriguing, their interpretation within the context of the study is not well articulated. In particular, the rationale for including cytosol is not sufficiently justified, given that ATP/GTP alone induces a pronounced effect, whereas cytosol alone does not.

Since the formation of anisosome requires HSP70, a cytosolic chaperone that likely needs to be imported into the nucleus, we included cytosol and ARS/GTP in our in vitro reaction. We revise the description in the result part to improve clarity (page 8-9).

(3) The authors should address why endogenous XPO1 does not co-localize with anisosomes, whereas overexpressed XPO1 does. This raises the possibility that the observed co-localization may be an artifact of non-physiological protein levels, which should be discussed.

As discussed in Yu H et al., Science 2021, proteins in anisosomes cannot be stained by antibodies due to an antibody accessibility issue. It was mentioned in our paper as “since antibody staining could not conclusively demonstrate the sequestration of endogenous XPO-1 in anisosomes due to an antibody penetration barrier {Yu, 2021 #937}.” We now revise this section completely to better clarify this point. We could see overexpressed XPO1 in anisosome because it has a mCherry tag.

(4) The iPSC-based model remains insufficiently characterized. While the authors propose that this system recapitulates the accumulation of liquid and solid aggregates resembling anisosomes, it is unclear whether this phenotype is robustly observed and whether KPT treatment effectively modulates it.

The full characterization of the iPSC-derived organoids is presented in a second paper that is posted in BioRxiv (https://www.biorxiv.org/content/10.1101/2025.11.09.687455v2), which is cited. This manuscript reports not only the accumulation of p-TDP43, but also other ALS-related phenotypes including cell death, gene transcriptional changes, cryptic exon inclusion etc. in mutant organoids.

(5) The rationale for the selected treatment durations is unclear, and the timing appears inconsistent across experiments (ranging from 3 to 16 hours), including within experiments involving the same compound. This variability should be justified or standardized.

The longer treatment (24 h) was used in the chemical genetic screen in which different drugs may act with different efficiency. To maximize our chance of detecting more drug effect, we used a longer treatment scheme. For later follow-up experiments involving Spuatin-1, Bortezomib, TRP, because the phenotype appears quickly. To avoid secondary effects from long treatment, we shortened the treatment to 3-5 hours. For LMB treatment, we used long treatment to reveal the steady-state phenotype (anisosome enlargement in size and reduction in number has reached maximum). This time point was determined in Figure 5A-C. In contrast, shorter treatment (5 h) was to reveal early changes that might be causal to the end-point phenotypes (e.g. the anisosome fusion phenotype could be detected as early as 5 h post-treatment). We have added some explanations in the result section to make this point clear.

(6) Several figure legends require clarification:

We thank the reviewer for pointing out the inconsistencies. We have corrected the outstanding issues, as explained below.

(a) In the section stating “Collectively, our results suggest that the stability and dynamics of anisosomes are modulated by XPO1-mediated nuclear export ...", the cited figure appears to be incorrect. This should refer to Figure 5L rather than Figure 5J.

Thanks for pointing out this error. This is now corrected.

(b) Figure 1B: Please specify the number of replicates per concentration, the number of cells analyzed, and the model used for regression analysis. Additionally, the legend indicates a treatment duration of 15 hours, whereas Figure 1A states 24 hours.

Due to the large sample size, each concentration was analyzed once. We have added other information to the figure legend. We also remove the redundant inaccurate information from the figure legend. The treatment time shown in the figure is correct as it was also indicated in the method.

(c) Figure 2G: The authors state "7 anisosomes per condition," but the graph displays only 4-6 data points. Please clarify what each data point represents.

We thank the reviewer for noticing the discrepancy and apologize for the error. We have corrected the figure legend to indicate that 4-6 anisosomes were analyzed for each condition. In Figure 2G, each data point represents the initial fluorescence loss rate averaged from the first 10 sec after reverse photobleaching.

(d) Figures 3B and 3G: Please clarify whether a defined threshold was used to determine a "reduction in anisosome number."

In Figure 3B, we used Z score >2 as the threshold. This is now mentioned in the legend and defined in the method. There is no Figure 3G.

(e) Figure 4B: These do not represent biological replicates, as all samples derive from a single cell line; rather, they constitute independent experimental replicates.

We have changed the figure legend throughout the paper accordingly.

(f) Figures 5B and 5H: The legend states "n = 3 biological repeats," but the number of data points shown appears higher. Please clarify.

In Figure 5B, the graph reflects data collected from 3 independent replicates. To ensure reliable baseline measurement, for each experiment, two independent control samples were included, which is why it has 6 data points. In Figure 5H, each dot represents a randomly selected imaging field. We now mention the total number of fields analyzed.

(g) Figures 5K, 6C, and 6E: "Mean Fluorescence Intensity (MPI)" should be corrected to "MFI."

These are all fixed. Thank you for pointing this out.

(h) Figure 6C: Please include the number of cells analyzed and provide relevant statistical measures (e.g., R^2^, p-value).

We now include the cell number in the legend and R^2^ and p-value in the figure.

(i) Figure 6D: The experimental timeline is unclear. Please specify the duration of incubation and the timing of each step.

We now revise the experimental scheme in Figure 6A to better explain the experiment and the sequence of different events. For Figure 6D, permeabilized cells were incubated with cytosol with or without ARS/GTP for 40 min. This information is added to the figure legend.

(j) Figure 7B: Improved labeling is needed (e.g., clarification of "mean spot volume") to better align with the figure legend.

To improve clarity, we change mean spot volume to p-TDP-43 puncta mean volume. This refers to the average volume of segmented phosphorylated TDP-43-positive puncta.

**Reviewer #3 (Public review):**

Summary:

TDP-43 proteinopathy is broadly found in neurodegenerative diseases. This manuscript investigates how nuclear export influences the biophysical properties of TDP-43. The authors use a combination of chemical screening and genome-wide siRNA screening to identify pathways that modulate TDP-43 liquid-to-solid transitions. Overall, the study employs a broad array of approaches and addresses an important question in TDP-43 pathobiology. The identification of nuclear export as a central regulator is compelling and conceptually aligns with the emerging view that TDP-43 nucleocytoplasmic trafficking is a major defect in neurodegeneration.

Strengths:

This work integrates chemical and genetic screening to identify novel modifiers. The candidates were validated in both reporter cell lines and iPS-differentiated organoids. The findings support the nucleocytoplasmic transport is important for the biophysical properties of TDP-43.

Comments on revised version.

The manuscript has been improved with more data and clarification. The RNase T1 treatment experiment suggests that RNA is required for anisosome integrity. However, this does not directly demonstrate LMB increases nuclear RNA availability as changes in protein composition or other RNA-dependent mechanisms may also contribute. The conclusion and discussion need to be edited to consider these alternative scenarios. Overall, as most of the evidence remains indirect, the manuscript should avoid overinterpretation regarding the mechanisms underlying TDP-43 phase transition and aggregation.

We thank the reviewer for this helpful suggestion. We have added a few sentences in the discussion (page 10) to acknowledge the limitation of the semi-permeabilized cell assay. Specifically, we mentioned that “However, our data does not exclude other RNA species or RNA-binding proteins as anisosome stabilizer. Whether RNA-dependent stabilization of anisosomes operates in the same way in intact cells also requires further validation.” We also revise our manuscript throughout to avoid over-interpretation.

**Recommendations for the authors:**

**Editor's notes:**

The value of the work is clear.

We also recognise that it may not be possible/practical to get around the 'incomplete' appellation attached to this body of work, by further experiments. However, there may be scope here to retreat from the less well supported mechanistic claims-by editing the title, abstract and discussion and thus earn a 'solid' descriptor on a revised paper that remains a useful addition.

The authors are best placed to consider creatively how to achieve this.

We thank the editors for this helpful suggestion. We have revised the manuscript extensively to address every single concerns of the reviewers.

**Reviewer #1 (Recommendations for the authors):**

This revision addresses some minor concerns and adds one mechanistic experiment of genuine value. However, the major deficiencies of the original submission persist: the exclusive reliance on non-physiological TDP-43 model systems without validation in more disease-relevant contexts, the unresolved asymmetry between the two XPO1 perturbation phenotypes, the thin organoid section that now has fewer readouts than before the revision, and overstatement of the mechanistic conclusions in the abstract and Discussion. The manuscript in its current form still does not provide methods, data, and analyses that sufficiently support the primary claim that nuclear export is an established key regulator of TDP-43 phase transitions with mechanistic and disease relevance.

As mentioned before, we have clarified the interpretation of the data, tempered conclusions where appropriate, and revised the text to explicitly acknowledge the limitations of the current study. We hope that these changes satisfactorily addressed the reviewer’s concern.

**Reviewer #2 (Recommendations for the authors):**

I would suggest adjusting the title to match the data, which shows so much more than just an effect of nuclear export.

We thank the reviewer for this suggestion. We have changed the title to “Cellular modifiers of TDP-43 phase transition and cytoplasmic aggregation”

When introducing the semi-permeabilized cell-based in vitro assay, it would be helpful to add a short statement describing what this model resembles, and what advantage it can bring to use this system in the context of the study.

We have revised this section extensively and hope that improves the clarity. See marked text in page 8-9.
